# Editorial Comment to Cystic partially differentiated nephroblastoma in a 74‐year‐old patient

**DOI:** 10.1002/iju5.12367

**Published:** 2021-08-25

**Authors:** Takeshi Yuasa

**Affiliations:** ^1^ Department of Urology Cancer Institute Hospital Japanese Foundation for Cancer Research Tokyo Japan

Wilms tumor is the most frequent tumor of the kidney in infants and children. Cystic partially differentiated nephroblastoma, which is a rare cystic variant of Wilms tumor (1%), is composed entirely of cysts, and their thin septa are the only solid portion of the tumor.^1^, ^2^ Compared to Wilms tumor, cystic partially differentiated nephroblastoma appears to have a better clinical outcome.^1^, ^2^ The recommended treatment strategies that have excellent outcomes are (i) surgery and (ii) surgery and adjuvant chemotherapy using vincristine and dactinomycin for Stage I and Stage II diseases, respectively.^1^, ^2^ Similar to Wilms tumor, cystic partially differentiated nephroblastoma typically presents in children. Adult cystic partially differentiated nephroblastoma is extremely rare, with only four cases reported to date.^3^, ^4^, ^5^

In this issue of *IJU Case Reports,* Hayashida et␣al. reported an adult case of cystic partially differentiated nephroblastoma.^3^ Various imaging studies, including ultrasonography, computed tomography, and magnetic resonance imaging, revealed a multilocular cystic tumor, which was classified as Bosniak IIF.^3^ As this tumor had not been previously identified on annual imaging study, it grew rapidly and was therefore considered a multilocular cystic renal cell cancer.^3^ The patient underwent radical nephrectomy and was diagnosed with cystic partially differentiated nephroblastoma by immunopathological examination. After surgical resection, no recurrence has occurred for the past 13 years.^3^

There are several important clinical messages in this case report. First, this is the oldest reported case (74 years old) and, given that previous cases were in their twenties to forties, this case demonstrates that cystic partially differentiated nephroblastoma can develop at any age.^3^ In addition, the surveillance period of this case was 13 years. This is the longest case in which no recurrence has occurred without any treatment. Therefore, we can confirm that surgical resection can be used as an effective treatment option in adult and pediatric cases. Although no recurrence of adult cystic partially differentiated nephroblastoma has been reported to date, the clinical characteristics may differ between pediatric and adult cases. Further accumulation of clinical information on adult cystic partially differentiated nephroblastoma is needed to determine the standard of care for this rare disease. Until this clinical information is acquired, clinicians should treat it as a potential malignant tumor.

## Conflict of interest

The author declares no conflict of interest.
